# Gynecology

**Published:** 1876-10

**Authors:** 


### II. Gynecology.

1. *Metastasis of Mumps in Women.* Damorest. (*Lyon Méd.*, No. 22.)

The author, in view of the recognized sympathy between the parotid glands and the genitalia, refers to the fact that, in women, metastasis occurs rather to the mammary and vulvar glands than to the ovaries, while in boys the testicles are affected. He reports two cases showing that the ovaries may be involved when females are attacked by mumps. Yet Trousseau, Grisolle and Niemeyer never noted such an occurrence; and Meyner, (*Gaz. Méd. de Lyon*, 1866,) publishes but one observation of the same. In the author's first case the parotiditis supplemented the menstrual flow, and the same was observed in the second case. In the latter, also, there was ovarian pain, and tenderness on both sides, with fever. Damorest concludes by remarking that it would be interesting to know whether, after such an attack, a young girl could become a mother.

2. *Formation of Vagina without employment of Cutting Instruments.* Le Fort. (*L'Union Méd.*, No. 91.)

A woman twenty-six years old, had suffered from general disorder at the menstural periods since her fifteenth year. In consequence of the absence of the vagina, the menses had been replaced by supplementary hæmorrhages; hæmoptyses, bleeding from the integument of the limbs, excessively painful and often intolerable epistaxis. In 1872 she entered La Pitié, where Labbé performed ten operations with the only result of creating a vulvar infundibulum a few centimetres in depth. Discouraged by this failure, the patient left the hospital after a sojourn there of eighteen months. But the pain and supplementary hæmorrhage continuing, she entered the Beaujon in July, 1875, where M. Anger succeeded LeFort, and performed the eleventh operation, which increased the depth of the infundibulum, but was followed by a severe attack of pelvi-peritonitis, which compelled LeFort to suspend interference until January, 1876.

He then operated by introducing a boxwood cylinder terminating in a metallic knob placed in connection with the positive pole of a battery of small elements, with sulphate of copper. The negative pole connected with a metallic disk surrounded by moistened linen and resting upon the abdominal surface. This weak current is not perceived by patients and only produces an eschar which is small in the immediate contact of the metallic rheophors. The apparatus was placed *in situ* every evening and kept there all night. Little by little the stem made a way for itself in the vesico-rectal septum, and, on the 26th of February it had penetrated as far as the cervix uteri. Then, for the first time, the patient experienced, at her menstrual epoch, a moderate flow of blood from the vagina; though the latter still escaped with difficulty as there was conjointly abdominal pain and slight hæmoptysis. But the treatment having been continued for another month, a canal was formed of sufficient size, and menstruation has since become painless, normal, and perfectly regular.

After two months stay at Vesinet, in consequence of a pneumonia with which she was attacked, the patient re-entered the Beaujon July 1st, and the treatment was renewed—this time for the purpose of giving to the vagina a sufficient size. Finally, on the 29th of July it was possible to establish, by the aid of the speculum, a small and irregular cervix ten centimeters in depth. An hysterometre introduced by the orifice of the neck, penetrated to the extent of six and a half centimeters, the uterine cavity being consequently of normal length. The result, therefore, is complete. In order to render it permanent, and to prevent the retraction of the artificially formed canal, it will suffice for the patient to introduce nightly an intra-vaginal pessary in the form of a cylindro-conical stem of box-wood or ivory—"that is," (adds the French author, with characteristic naiveté,) "in default of those physiological measures which her years might permit."

3. *On the Treatment of Uterine Affections by Hydrotherapy.* DERIVAUX. (*Thése de Paris*, No. 190.)

Here are the author's conclusions upon the employment of cold water in uterine disorders:

1. Hydrotherapy should be regarded as the basis of treatment in both the first and second stages of chronic parenchymatous metritis. With it should be often associated the employment of the actual cautery. It has no direct curative action upon ulcerative folliculitis, but hastens the cicatrization of ulcerations, caused by uterine engorgement.

2. Hydrotherapy is a most useful and necessary auxiliary in the treatment of chronic metritis involving the mucous membrane. By revulsion and derivation, it overcomes the two most serious symptoms of the disease, viz., metrorrhagia and uterine leucorrhœa. Its efficacy in hæmorrhage renders it a precious palliative in the gravest affections to which the uterus is liable — fibromata and carcinomata.

3. By its general restorative action and its local tonic effect upon the suspensory ligaments, hydrotherapy is always more or less remedial in hysteroptosis, and relieves its most painful sequelæ. When anteversion is caused by uterine engorgement, it procures reposition of the organ. But its action is most feeble in retroversion, and quite *nul* in uterine inflexion.

4. Hydrotherapy constitutes the most efficacious treatment in amenorrhœa from constitutional feebleness, and dysmenorrhœa from a similar cause. In menorrhagia it takes precedence of all other therapeutic agents, and combats with success congestive and neuralgic dysmenorrhœas, and the disorders attending the menopause.

5. General and local nervous disorders, and the painful symptoms connected with uterine lesions, disappear under the influence of hydrotherapy even before the material local disorder is profoundly modified. The restoration of the general health is obtained by no medication with as much sureness and rapidity as by cold applications. And it may be added that the most advanced degree of marasmus will not permit us to think of any other therapeutic agent.

